# UCP2 Inhibits ROS-Mediated Apoptosis in A549 under Hypoxic Conditions

**DOI:** 10.1371/journal.pone.0030714

**Published:** 2012-01-24

**Authors:** Sanming Deng, Ye Yang, Yong Han, Xiaofei Li, Xiaoping Wang, Xueyong Li, Zhipei Zhang, Yunjie Wang

**Affiliations:** 1 Department of Thoracic Surgery, Tangdu Hospital, The Fourth Military Medical University, Xi'an, Shaanxi Province, China; 2 Department of Thoracic Surgery, The Hospital of Medical College of Chinese People's Armed Police Force, Tianjin, China; 3 Department of Thoracic Surgery, Shaanxi Province People's Hospital, Xi'an, Shaanxi Province, China; 4 Department of Plastics and Burns, Tangdu Hospital, The Fourth Military Medical University, Xi'an, Shaanxi Province, China; University of Kentucky College of Medicine, United States of America

## Abstract

The Crosstalk between a tumor and its hypoxic microenvironment has become increasingly important. However, the exact role of UCP2 function in cancer cells under hypoxia remains unknown. In this study, UCP2 showed anti-apoptotic properties in A549 cells under hypoxic conditions. Over-expression of UCP2 in A549 cells inhibited reactive oxygen species (ROS) accumulation (P<0.001) and apoptosis (P<0.001) compared to the controls when the cells were exposed to hypoxia. Moreover, over-expression of UCP2 inhibited the release of cytochrome C and reduced the activation of caspase-9. Conversely, suppression of UCP2 resulted in the ROS generation (P = 0.006), the induction of apoptosis (P<0.001), and the release of cytochrome C from mitochondria to the cytosolic fraction, thus activating caspase-9. These data suggest that over-expression of UCP2 has anti-apoptotic properties by inhibiting ROS-mediated apoptosis in A549 cells under hypoxic conditions.

## Introduction

UCP2 belongs to a family of anion carrier proteins and is expressed in the inner membrane of the mitochondria [1]. Although UCP2 over-expression has been described in various types of cancer, including human colon cancer cells [2], thyroid tumor [3], and hepatocellular carcinoma [4], the precise function of UCP2 in cells remains unknown [1]. It is widely accepted that UCP2 can be activated by reactive oxygen species (ROS), forming part of a negative feedback mechanism that mitigates excessive ROS production [5]–[8].

Abundant evidence has shown that ROS are not only the inevitable by-products of oxygen metabolism, but also play a role in cellular signaling in tumors cells [9]–[12]. In HCT116, HT29 and HepG2, etc., over-expression of UCP2 plays an anti-apoptotic role by modulating the generation of intracellular ROS after tumor cells are exposed to chemotherapeutic agents [7], [13], [14]. Meanwhile,Zoltan Derdak et al [7] also demonstrated that UCP2 expression in HCT116 human colon cancer cells decreased apoptosis induced by mechanisms involving modulation of p53 pathway. Thus, UCP2 may play an integral role in the adaptive response of cancer cells to chemotherapeutics. Hypoxia is a well-characterized parameter of the tumor microenvironment that profoundly influences cancer progression and its response to treatment [15]. Hypoxia in cancer cells also triggers an increase in mitochondrial ROS production [16], [17]. Therefore, we hypothesize that UCP2 may play a role in tumor cells in response to hypoxic stimuli.

Collins et al. [4] showed that increased UCP2 expression reduced apoptosis and ROS in response to the oxidative stress induced by hypoxia/re-oxygenation in HepG2 cells. However, they were unable to identify the mechanism underlying this phenomenon. The authors presumed that increased UCP2 activity may decrease the generation of intracellular ROS in mitochondria and stabilize the membrane, making the cell more resistant to apoptosis. A549 cells, derived from a lung adenocarcinoma, were chosen for the current study. To establish hypoxia, the cells were cultured in serum-free Dulbecco's modified Eagle's medium (DMEM) with CoCl_2_ under standard culture conditions for 24 hrs. The UCP2 levels were then regulated by over-expression or knocked down by small interfering RNA (siRNA) in A549 cells to confirm the function of UCP2.

## Materials and Methods

### Cell culture and induction of a hypoxic environment

The lung adenocarcinoma cell line A549 was chosen for this study. A549 cells were obtained from the American Type Culture Collection (Manassas, VA) and cultured in DMEM supplemented with 10% fetal bovine serum (FBS), penicillin 100,000 U/l and streptomycin 100 mg/l. To establish a hypoxic condition, the cells were cultured in serum-free DMEM with 100 µM CoCl_2_ (Sigma, USA), 150 µM CoCl_2_ or 200 µM CoCl_2_ under standard culture conditions (5% CO_2_ and 37°C) for 24 hrs. In the following experiments performed in this study for detecting the function of UCP2, hypoxic condition was induced using 150 µM CoCl_2_ with serum-free DMEM for 24 hrs.

### Assessment of apoptosis

The percentage of apoptotic cells was determined by monitoring the translocation of phosphatidylserine to the cell surface using an Annexin V-FITC apoptosis detection kit (Sigma, UK) according to the manufacturer's instructions. Cells were evaluated for apoptosis using a FACSCalibur flow cytometer (BD Biosciences) with Annexin V–FITC and PI double staining. Fluorescence was measured with an excitation wavelength of 480 nm through FL-1 (530 nm) and FL-2 filters (585 nm).

### Plasmids and cell transfection

In UCP2 over-expression experiments, human skeletal muscle total RNA was reverse transcribed, and full-length human UCP2 cDNA was amplified by PCR with sequence-specific primers. The double-digested cDNA was then inserted into pcDNA 3.1/Zeo (-) (sites BamHI/EcoRI) using the rapid DNA ligation kit (Roche, USA). The siRNA against human UCP2 was chemically synthesized by GenePharma (Shanghai, China) as the following oligonucleotide sequences: sense: 5′-GCACCGUCAAUGCCUACAATT-3′, antisense: 5′-UUGUAGGCAUUGACGGUGCTT-3′. Transient transfection of the UCP2 plasmid and siRNA were performed with Lipofectamine 2000 (Invitrogen, Carlsbad, CA). A total of 5 µg of plasmid or 100 pmol of siRNA containing Lipofectamine was applied in a final volume of 0.75 ml per well in 6-well plates.

### Western blotting

The cells were homogenized in a lysis buffer containing 8 M urea, 10% SDS, 1 M DTT, and protease inhibitors. Protein concentrations were determined using a BCA Protein Assay Reagent Kit (Beyotime, China). The cytosolic fraction was isolated from cells as described by Johnson [18] and Catherine Plin [19]. The levels of UCP2, cytochrome C, and caspase-9 protein were quantified by Western blotting using the indicated antibodies in cytosolic fraction. The primary antibody to cytochrome C was used at a concentration of 1∶500 (Abcam, UK), caspase-9 (1∶500; Abcam, UK), and UCP2 (1∶500; Abcam, UK). A mouse monoclonal anti-β-actin antibody (Sigma) was used for normalization. Secondary antibodies were conjugated with horseradish peroxidase, and the signals were detected using ECL reagent (GE Healthcare, USA).

### Real-time PCR

At the commencement of each experiment, cells were harvested at the indicated time points for the extraction of RNA, and 2 µg of total RNA was reverse transcribed into cDNA (TRizol, Invitrogen; MLV Reverse transcriptase, Promega). To measure mRNA expression of UCP2, one-step RT-PCR using the SYBR Green PCR Master Mix kit (Applied Biosystems) was used according to the manufacturer's instructions. The following primers were used: UCP2, forward: 5′- GTCGGAGATACCAAAGCAC -3′, reverse: 5′- ATGGCATTACGAGCAACA -3′. β-actin was used as an internal reference.

### ROS measurement

The generation of intracellular ROS was examined by flow cytometry using an ROS assay kit (Applygen Co. Ltd., China). Cells (1×10^5^) were incubated with 10 µM DCFH-DA in complete medium for 30 min at 37°C to allow cellular incorporation. The cells were then washed and resuspended in 1 mL of PBS. Single cells were then analyzed by flow cytometry (BD Biosciences) with an emission wavelength of 502 nm and an excitation wavelength of 530 nm. A total of 10,000 cells were counted per sample.

### Statistical analysis

Data are expressed as the mean ± standard error (SE). The SPSS 16.0 software package was used for statistical analysis, and Student's *t* test was applied for comparison of the means of the two groups of experimental data. An analysis of variance (ANOVA) was applied to compare the means of multiple groups of measurement data. Significance was defined as P<0.05.

## Results

### The relationship between the expression of UCP2 and HIF-1α accumulation at different levels of hypoxia in vitro

**Figure 1:**
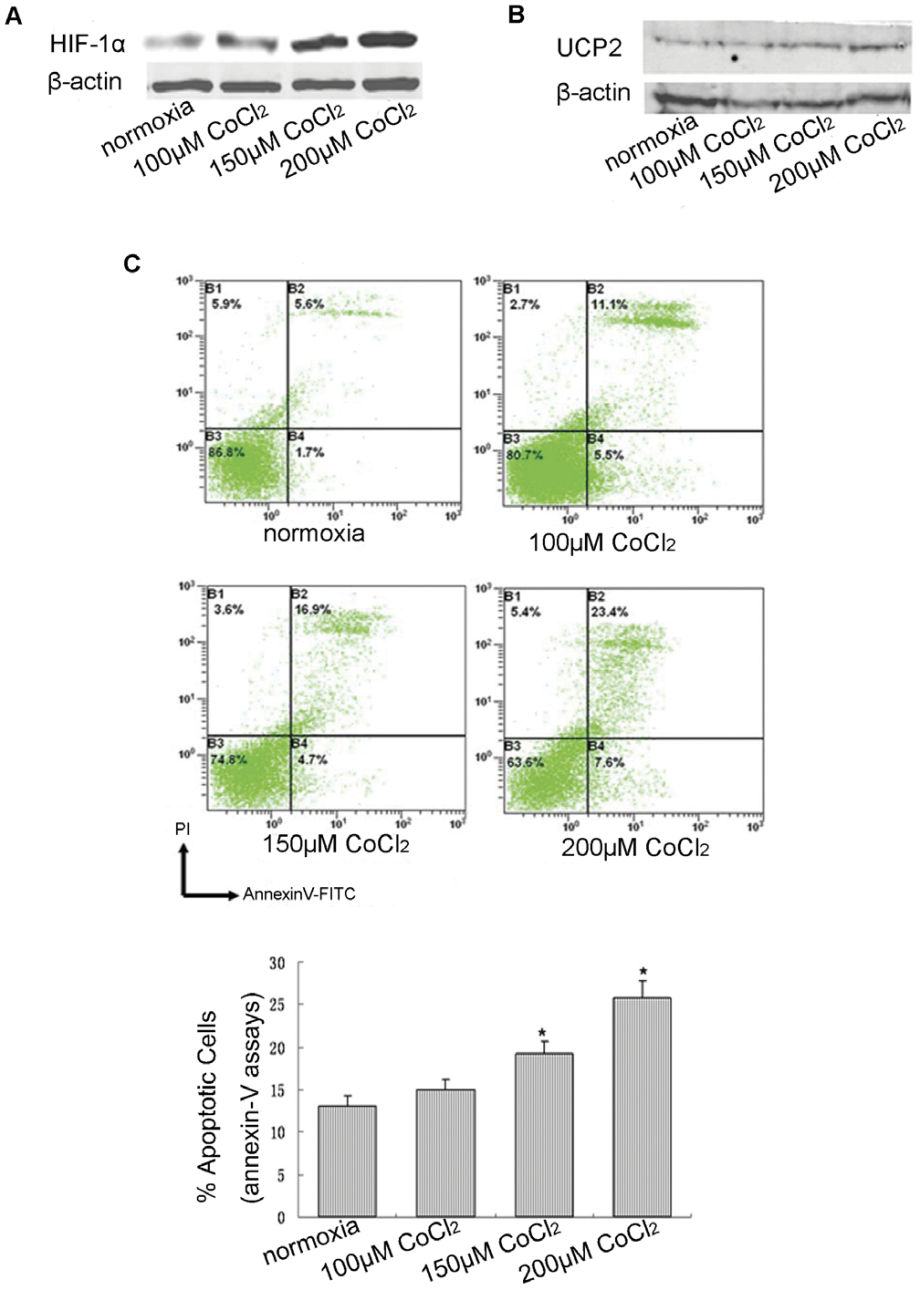
A549 cells were cultured in serum-free DMEM under the following conditions: normoxia, 100 µM CoCl_2_, 150 µM CoCl_2_, or 200 µM CoCl_2_ for 24 hrs. A: Western blot analysis of HIF-1α in A549 cells under different levels of hypoxia (n = 3). B: Western blot analysis of UCP2 in A549 cells under different levels of hypoxia (n = 3). C: The relationship between apoptosis and hypoxia in A549 cells. Data are mean ± SEM (n = 5). *P<0.05, compared with normoxic conditions.

Hypoxia can be generated by oxygen deprivation or by the addition of CoCl_2_ [20]. Hypoxia was induced by adding CoCl_2_ to serum-free DMEM for 24 hrs. As seen in Figure 1A, the expression of HIF-1α gradually increased as the oxygen level decreased, confirming that hypoxia could be generated by the addition of CoCl_2_. The protein expression level of UCP2 was detected by Western blot, and UCP2 expression increased in a dose-dependent manner that was correlated to the extent of hypoxia observed in vitro (Figure 1B).

### The relationship between apoptosis and different levels of hypoxia in vitro

When being exposed exposure to hypoxia at three different concentrations of CoCl_2_, cancer cells showed a dose-dependent increase in apoptosis based on the Annexin V–PI binding assay. Compared to the normoxic condition, the amount of apoptosis significantly increased following the addition of 150 µM CoCl_2_ (P = 0.032; Figure 1C).

### Over-expression or knockdown of UCP2 via transfection with over-expression plasmid or siRNA

**Figure 2:**
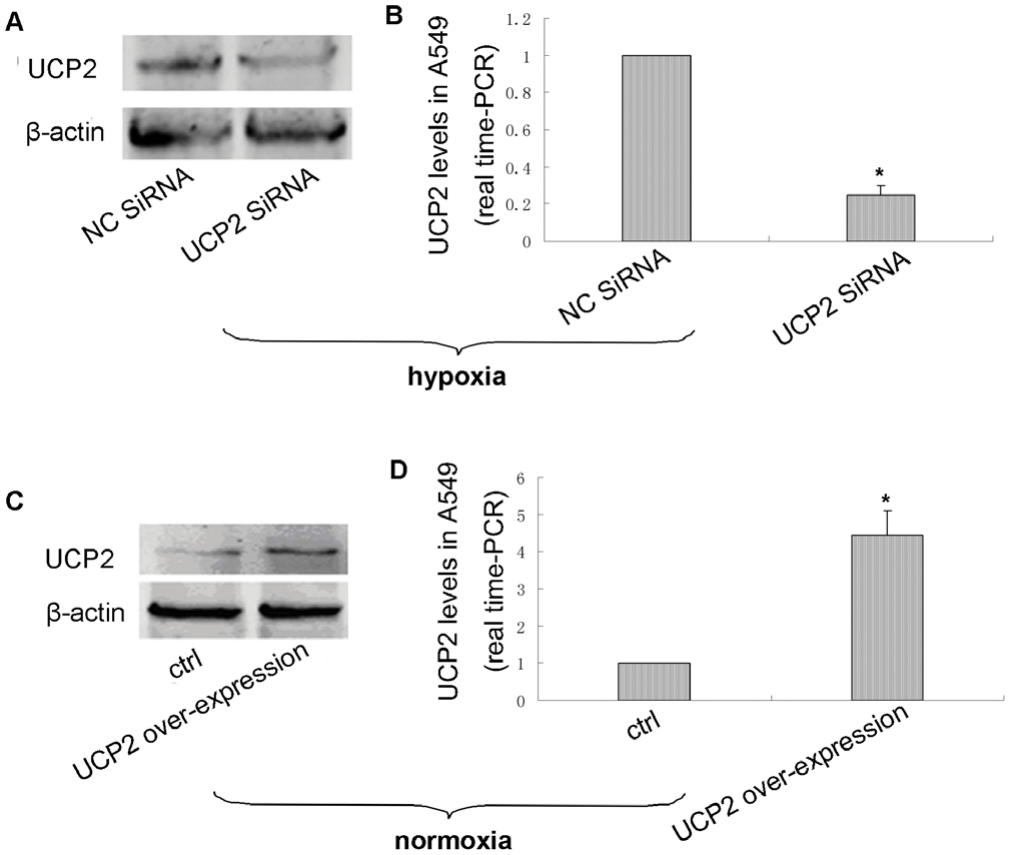
UCP2 levels are regulated by UCP2 expression. Data are mean ± SEM. *P<0.05,compared with negative control cells. NC siRNA: negative control siRNA-transfected cells; UCP2 siRNA: UCP2 siRNA-transfected cells; Ctrl: negative control pcDNA3.1-transfected cells; UCP2 over-expression: pcDNA3.1-UCP2-transfected cells. A: UCP2 protein levels in UCP2 siRNA-transfected cells under hypoxic conditions (n = 3). B: Concentration of UCP2 mRNA in UCP2 siRNA-transfected cells under hypoxic conditions (n = 5). C: UCP2 protein levels in cells with over-expressed UCP2 under normoxic conditions (n = 3). D. Concentration of UCP2 mRNA in cells with over-expressed UCP2 under normoxic conditions (n = 5).

Given that UCP2 protein expression is very low under normoxic conditions, we evaluated the function of UCP2 siRNA under hypoxia. Under these conditions, UCP2 mRNA concentration and protein expression was significantly reduced in UCP2-siRNA-transfected A549 cells compared to the controls (P<0.001; Figure 2 A and B). Thus, the siRNA resulted in the potent inhibition of UCP2 expression in A549 cells under hypoxic conditions. To validate the over-expression, UCP2 was detected by Western blotting and real-time PCR under normoxic conditions. UCP2 mRNA concentrations and protein expression levels were significantly increased compared to that of pcDNA 3.1-control-transfected cells (P<0.001; Figure 2 C and D).

### The relationship between over-expressed UCP2 and anti-apoptotic function

**Figure 3:**
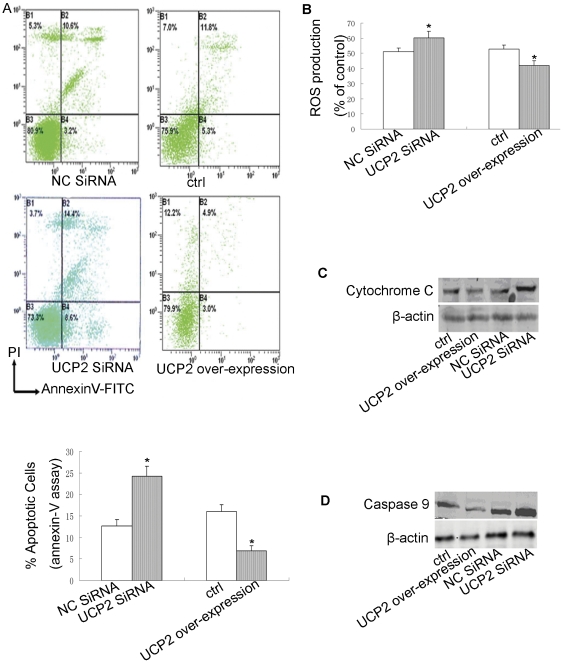
Altered UCP2 expression and apoptosis. Date are mean ± SEM. *P<0.05, compared with negative control cells. NC siRNA: negative control siRNA-transfected cells; UCP2 siRNA: UCP2 siRNA transfected cells; Ctrl: negative control pcDNA3.1-transfected cells; UCP2 over-expression: pcDNA3.1-UCP2-transfected cells. A: Apoptosis is detected by Annexin V–FITC/PI staining(n = 5). B: The levels of ROS in A549 cells with over-expressed or suppressed UCP2 under hypoxic conditions(n = 5). C: Cytochrome C in cytosolic fraction (n = 3). D: Levels of the activating caspase-9 (n = 3).

Cellular apoptosis was detected by Annexin V–FITC/PI staining. Knockdown of UCP2 under hypoxic conditions significantly increased apoptosis compared to the control-siRNA-treated cells (P<0.001; Figure 3 A). Conversely, under the same hypoxic conditions, over-expression of UCP2 significantly decreased the level of apoptosis (P<0.001; Figure 3 A).

Increasing evidence has shown that UCP2 is activated by ROS and mitigates excessive ROS production in the form of a negative feedback mechanism [5]–[8]. Therefore, the levels of ROS were detected in A549 cells with over-expressed or suppressed UCP2 under hypoxic conditions. Intracellular ROS levels were significantly increased in cells with suppressed UCP2 compared to the control cells (P = 0.006), while ROS levels in cells with over-expressed UCP2 were lower than that of the control cells under the same hypoxic conditions (P<0.001; Figure 3B).

The mitochondrial dysfunction associated with ROS-mediated cell death induced the activation of pro-apoptotic proteins such as cytochrome C, which was released from the mitochondria into the cytosol [21] and led to caspase-dependent or caspase-independent apoptosis. in UCP2-siRNA-transfected A549 cells, which are known to have very little UCP2 protein under hypoxic conditions, the amount of cytosolic cytochrome C was significantly increased (Figure 3 C). Cytochrome C activated several members of the caspase family, most prominently the initiator, caspase-9. We found that caspase-9 expression was increased in A549 cells compared to the controls (Figure 3D). Furthermore, over-expression of UCP2 inhibited the release of cytochrome C (Figure 3C) and reduced the activation of caspase-9 (Figure 3 D). These results suggest that UCP2 could regulate the release of cytochrome C and the activation of caspase-9.

## Discussion

UCP2 belongs to a family of anion carrier proteins and is expressed in the inner membrane of mitochondria [1]. Numerous studies have indicated that UCP2 expression is upregulated in several tumor types [2]–[4]. Immunohistochemical studies using clinical tissue microarrays demonstrated that the frequency and intensity of UCP2 staining was correlated with the degree of neoplastic changes in the colon. Although UCP2 was rarely detected in hyperplastic polyps (11%), it was present in tubular adenomas (58.8%) and colon adenocarcinomas (86.0%) [2].

Hypoxia is a well-characterized parameter of the tumor microenvironment that profoundly influences cancer progression and its response to treatment [15]. In addition, hypoxia may control the expression of several target genes involved in many aspects of cancer progression, including angiogenesis [22], [23], chemoresistance [24], [25], apoptosis [26], [27], invasion [28], [29], and metastasis [30].

Hypoxia is generated by either oxygen deprivation or the addition of CoCl_2_ [20] to the cell medium. Here, hypoxia was induced by adding CoCl_2_ to serum-free DMEM for 24 hrs. Our results demonstrated that the expression of HIF-1α gradually increased in correlation with the decreasing oxygen levels, confirming that hypoxia was generated by the addition of CoCl_2_.

Hypoxia can inhibit apoptosis in cancer cells. Olga Karovic et al. [20] showed that the toxic effects of cobalt in cells resemble that of hypoxia. Moreover, they found no significant increase in apoptosis when cells cultured with 10% FBS were exposed to 200 µM CoCl_2_, whereas cells exposed to CoCl_2_ (500 µM) exhibited typical features of cell death by apoptosis. Our results indicated that A549 cells exposed to 100 µM CoCl_2_ for 24 hrs showed minor signs of cytotoxicity. However, compared to the cells cultured under normoxic conditions, no significant increase in the percentage of apoptotic cells was observed. In addition, the amount of apoptosis resulting from the addition of 150 µM CoCl_2_ under hypoxic conditions was different from the cells cultured in normoxic conditions. Although our results are similar to those of Olga Karovic et al. [20], there was a difference in the concentration of CoCl_2_, which induced typical features of cell death by apoptosis. This result may be due to the use of the serum-free DMEM under hypoxic conditions in the present study, as Olga Karovic et al. used DMEM with serum.

The expression of UCP2 showed a dose-dependent increase under hypoxic conditions. Given that UCP2 protein expression was weak under normoxic conditions, we detected the function of UCP2 siRNA under hypoxia. Compared to that of the negative control siRNA-treated cells under hypoxic conditions, UCP2 mRNA concentration and protein expression were decreased following UCP2 knockdown in A549 cells. The expression of UCP2 mRNA and protein increased compared to control cells, indicating that such a strategy allowed the potent regulation of UCP2 expression in A549 cells.

The importance of crosstalk between a type of cancer and its hypoxic microenvironment has become increasingly recognized. However, little is known regarding the precise role of UCP2 in cancer cells under hypoxic conditions. To confirm the function of UCP2 under hypoxia, its expression was modulated in A549 cells under hypoxic conditions. We found that the level of apoptosis significantly increased following knockdown of UCP2 compared with that of the control cells, whereas the level of apoptosis decreased in response to UCP2 over-expression. Collins et al. [4] proved that increased expression of UCP2 reduced apoptosis and ROS in response to oxidative stress induced by hypoxia/re-oxygenation in HepG2 cells. In their study, however, the pathway mediating this phenomenon was not clear. They presumed that increased UCP2 activity may decrease the generation of intracellular ROS mitochondria and stabilize the membrane, making it more resistant to apoptosis.

UCP2 is a member of a family of anion carrier proteins expressed in the inner membrane of the mitochondria, in which the primary function is to allow the reentry of protons to the mitochondrial matrix by dissipating the proton gradient and subsequently decreasing ROS production [1], [5], [6], [8]. It was previously demonstrated that the main function of UCP2 was to regulate by ROS production in several tissue types [31]–[34]. In cancer cells, UCP2 may play an integral role in the adaptive response to chemotherapeutics [7], [13], [14]. A drug-resistant subset of cancer cells derived from leukemia, melanoma, and colon cancer cells exhibited increased levels of UCP2 and diminished susceptibility to cytotoxic effects [5], [7], [14], [35]. In addition, in drug-sensitive HL-60 cells (colon), UCP2 prevented ROS-induced impairments in mitochondrial metabolism [14]. Francisca M. et al [13] demonstrated that inhibition of UCP2 resulted in a marked increase in the rate of mitochondrial ROS production and caused cytotoxicity after exposure of colon cancer cells to cisplatin. Consistent with our results, previous studies have shown that the main function of UCP2 in cancer cells is to regulate ROS production. We found that intracellular ROS was significantly higher in cells with suppressed UCP2 compared to control cells, and ROS generation in cells with over-expressed UCP2 was lower than that of control cells under the same hypoxic conditions.

There is abundant evidence showing that the ROS are not only the inevitable byproducts of oxygen metabolism, and they also play a role in cellular signaling in several types of tumors [9]–[12]. Overproduction or accumulation of ROS decreases mitochondrial membrane potentials and leads to the swelling and disruption of mitochondria. Cytochrome C release could activate the family of caspase proteins that lead to apoptosis [9]–[12]. Furthermore, Jee-Youn Kim et al. [12] demonstrated that caspase-9 can be activated by ROS without the involvement of cytochrome C release. Our findings support the hypothesis that ROS is an important mechanism mediating the apoptotic pathway, and our results show the presence of high levels of cytochrome C and ROS in the cytosol of cells lacking UCP2. The release of cytochrome C could activate caspase-9 and increase apoptosis. Here, we found that over-expression of UCP2 decreased intracellular ROS, inhibited the release of cytochrome C, and decreased caspase-9 activation, resulting in cell survival under hypoxic conditions. Other data [7] proved that UCP2 expression in HCT116 human colon cancer cells decreased apoptosis induced by mechanisms involving modulation of p53 pathway, a pivotal tumor suppressor, but it still needs other experiments to prove whether UCP2 plays anti-apoptotic role by modulation of p53 pathway in hypoxia.

The results of this study indicated that over-expression of UCP2 has anti-apoptotic effects by inhibiting ROS-mediated apoptosis in A549 cells under hypoxic conditions. Therefore, UCP2 may provide a new target for the treatment of cancer cells under hypoxia. Given that hypoxia is a normal state in tumors, its role as an anti-apoptotic mechanism in cancer is supported by our findings.
